# Prognostic significance of pathologic nodal positivity in non-metastatic patients with renal cell carcinoma who underwent radical or partial nephrectomy

**DOI:** 10.1038/s41598-021-82750-y

**Published:** 2021-02-04

**Authors:** Sung Han Kim, Boram Park, Eu Chang Hwang, Sung-Hoo Hong, Chang Wook Jeong, Cheol Kwak, Seok Soo Byun, Jinsoo Chung

**Affiliations:** 1 Department of Urology, Center for Urologic Cancer, Research Institute and Hospital of National Cancer Center, 323 Ilsan-ro, Ilsandong-gu, Goyang-si, Gyeonggi-do 10408 Republic of Korea; 2 Statistics and Data Center, Research Institute for Future Medicine, Samsung Medical Center, Seoul, Republic of Korea; 3 Department of Urology, Chonnam National University Medical School, Hwasun, Republic of Korea; 4 Department of Urology, Seoul St. Mary’s Hospital, The Catholic University of Korea, Seoul, Republic of Korea; 5 Department of Urology, Seoul National University College of Medicine and Hospital, Seoul, Republic of Korea; 6 Department of Urology, Seoul National University Bundang Hospital, Seongnam, Republic of Korea

**Keywords:** Cancer, Biomarkers, Health care, Medical research, Nephrology, Oncology, Pathogenesis, Risk factors, Signs and symptoms, Urology

## Abstract

This retrospective, five-multicenter study was aimed to evaluate the prognostic impact of pathologic nodal positivity on recurrence-free (RFS), metastasis-free (MFS), overall (OS), and cancer-specific (CSS) survivals in patients with non-metastatic renal cell carcinoma (nmRCC) who underwent either radical or partial nephrectomy with/without LN dissection. A total of 4236 nmRCC patients was enrolled between 2000 and 2012, and followed up through the end of 2017. Survival measures were compared between 52 (1.2%) stage pT1-4N1 (LN+) patients and 4184 (98.8%) stage pT1-4N0 (LN−) patients using Kaplan–Meier analysis with the log-rank test and Cox regression analysis to determine the prognostic risk factors for each survival measure. During the median 43.8-month follow-up, 410 (9.7%) recurrences, 141 (3.3%) metastases, and 351 (8.3%) deaths, including 212 (5.0%) cancer-specific deaths, were reported. The risk factor analyses showed that predictive factors for RFS, CSS, and OS were similar, whereas those of MFS were not. After adjusting for significant clinical factors affecting survival outcomes considering the hazard ratios (HR) of each group, the LN+ group, even those with low pT stage, had similar to or worse survival outcomes than the pT3N0 (LN−) group in multivariable analysis and had significantly more relationship with RFS than MFS. All survival measures were significantly worse in pT1-2N1 patients (MFS/RFS/OS/CSS; HR 4.12/HR 3.19/HR 4.41/HR 7.22) than in pT3-4N0 patients (HR 3.08/HR 2.92/HR 2.09/HR 3.73). Therefore, LN+ had an impact on survival outcomes worse than pT3-4N0 and significantly affected local recurrence rather than distant metastasis compared to LN− in nmRCC after radical or partial nephrectomy.

## Introduction

The current standard of care for the organ-confined renal cell carcinoma (RCC) is curative nephrectomy either radically or partially of the primary kidney tumor^1,2^. About one-third of surgical patients experience either local recurrence or distant metastasis via lymphatic or hematogenous systems postoperatively, with a 20–40% 5-year recurrence-free survival (RFS) rate and 5–15% 5-year metastasis-free survival (MFS) rate^2,3^. Several high risk factors for local recurrence and distant metastasis have been defined from many previous papers in non-metastatic RCC (nmRCC) after curative nephrectomy, such as pathological/clinical stage of nodal status and primary tumor, tumor nuclear grade, treatment-free interval, or disease-free survival^4–6^.

For those high-risk factors, researchers have discussed about the necessity and the potential benefit of adjuvant therapies after surgery to prevent local recurrence or distant metastasis resulting in several trials, which have not successfully proven its primary goal of improving survivals, except in 5–10% of patients expecting a favorable improvement of survivals with selected indications^7,8^. Potential reasons for the failed clinical trial of adjuvant therapy after curative nephrectomy included the absence of proven efficacious adjuvant agents, no true indicative risk factors for patients receiving the adjuvant agents, the absence of targeted lesions on postoperative imaging studies during the follow-up, a non-standardized and stratified strategy for local recurrence and distant metastasis, and a heterogenous group of patients with different tumor burdens at the time of nephrectomy^9^.

Among the diverse indications of high risk of either local recurrence or distant metastasis after curative nephrectomy in nmRCC, the prognostic significance of nodal status on survival and the necessity of LN dissection during the nephrectomy have been always in great debates even though LN dissection has not recommended in routine nephrectomy for nmRCC after the meta-analytic reports indicating an insignificant prognostic benefit from LN dissection on survival in both nmRCC and mRCC^10^. However, LN dissection has still been performed in less than 20% of cases where there is suspicion of nodal positivity either preoperatively or intraoperatively, because the additional pathologic information provided by LN dissection is useful for further therapeutic planning^3,11–14^ and cancer-specific survival (CSS) was significantly associated with the extent of LND, the total number of removed LN, and the different staged LND, especially for patients with either a sarcomatoid component or large tumor size^15,16^.

Therefore, this retrospective multicenter study enrolled over 4000 patients with nmRCC who underwent either radical or partial nephrectomy at five tertiary Korean institutions to evaluate the prognostic impact of pathologic nodal positivity (LN+) on RFS, MFS, OS, and CSS in comparison to non-nodal positivity (LN−) and to search for any potential role of LN dissection in stratified T stages proving information about further indications for the successful systemic adjuvant therapy to prevent from either local recurrence or distant metastasis.

## Patients and methods

### Ethics statement

Following approval of this retrospective multicenter study of the previously approved nephrectomized RCC multicentric database by the Institutional Review Board of the National Cancer Center (IRB No. NCC 2018-0045 and B1202/145-102), the IRB approved all exemptions from written consent. This study was conducted in accordance with the Declaration of Helsinki. All the database was anonymized before the statistical analyses.

### Patient criteria

From the 2002 though 2012, overlapping data for patients at five institutions from two Korean multicentric RCC databases was extracted for this study, and follow-up data, including recurrence, metastasis and survival outcomes, was obtained until the end of 2017. One Korean multicentric database was organ-confined nmRCC (named as KORCC DB) and comprised of 6000 Korean patients who underwent either radical or partial nephrectomy at seven Korean tertiary institutions, and the other multicentric RCC database was a Korean metastatic RCC (mRCC) database of 6849 patients from 13 tertiary academic centers^17,18^. A total of the 4236 patients were finally enrolled from the five overlapping insitutions from two multicentric RCC databases. The five institutions were Chonnam National University Hwasun Hospital, the National Cancer Center, Seoul National University Bundang Hospital, Seoul National University Hospital and Seoul St. Mary’s Hospital.

The exclusion criteria for this study were postoperative recurrence within 3 months, age < 19 years-old at the time of nephrectomy, with benign histology, nephrectomy for cytoreductive purposes, intraoperative death or postoperative death within 1 month, and lack of follow-up records. Included patients underwent curative partial or radical nephrectomy with/without LN dissection. LN dissection was performed if preoperative nodal positivity sized > 1.0 cm was observed in the preoperative imaging studies or intraoperatively, prominently enlarged or palpable LN suspected of nodal positivity without indications was observed at preoperative imaging studies. All decisions regarding surgical procedure and performance of LN dissection were made at the surgeon’s discretion. There was no standardized nephrectomy procedure established at the beginning of the database enrollment^17,18^. However, information about partial and radical nephrectomy procedures has been documented in previously published papers for Korean Renal Cell Carcinoma Research (KORCC) group^17^. The risk parameters analyzed in this study included baseline anthropometric characteristics, laboratory and pathologic data, and survival prognoses including RFS, MFS, OS, and CSS. RFS and MFS were defined as local recurrence without any metastasis and distant metastasis without local recurrence 3 months or more postoperatively, respectively.

### Statistical analysis

Frequencies (percentages) for categorical variables and mean ± standard deviation (STD) or median (interquartile range, IQR) for continuous variables were summarized. Differences between pT1-4N1 patients (the pathologic nodal positive or LN+ group) and T1-4N0 patients (the nodal negative or LN− group) were evaluated using the *t* test or Wilcoxon rank-sum test for continuous variables and Fisher’s exact test or Pearson’s chi-square test for categorical variables as appropriate. Recurrence at the operative field was diagnosed with imaging studies. Recurrence occurring within 3 months after nephrectomy was classified as synchronous mRCC rather than postoperative local recurrence. Survival curves were estimated using the Kaplan–Meier method, and differences according to group in RFS, MFS, OS and CSS were tested by log-rank test. The Cox proportional hazards model was applied to find the prognostic risk factors in RFS, MFS, OS and CSS, and presented as a hazard ratio (HR) and 95% confidence interval (CI). Some additional sub-analyses were performed to compare the survival according to the nodal states among patients with only LN dissection and those staged pT2-4 with and without LN dissection. We reported statistically significant when two-sided *p* values were < 0.05. All results were analyzed using SAS 9.4 software (SAS Institute, Inc., Cary, NC, USA) and R software, version 3.5.0 (R Project for Statistical Computing).

## Results

### Baseline Characteristics

The baseline characteristics are shown in Table 1 including a comparison of baseline characteristics between the LN+ (n = 52, 1.2%) and LN− (n = 4184, 98.8%) groups. There were 410 (9.7%) recurrences, 141 (3.3%) metastases and 351 (8.3%) deaths, including 212 (5.0%) cancer-specific deaths after curative nephrectomy during a 43.8 (range 1.0–293.1) months of median follow-up. The significant differences between LN− and LN+ groups showed in body mass index; preoperative laboratory findings including hemoglobin, platelet count, albumin, hepatic enzymes (AST and ALT) levels; type of nephrectomy performed, operative extent; and pathological characteristics including T stage, nuclear grade, histology and the presence of sarcomatoid differentiation, tumor necrosis, lymphovascular invasion, and capsule invasion (*p* < 0.05, Table 1).

**Table 1:** Baseline characteristics (N = 4236).

		Total	pTxN1	pT1-4N0	*p* value
		(n = 4236)	(n = 52)	(n = 4184)	
Follow-up duration	Median (range)	43.79 (1.02–293.06)	82.52 (1.35–136.24)	43.69 (1.02–293.06)	
Age at operation	Mean ± STD	55.53 ± 12.42	53.56 ± 13.85	55.56 ± 12.41	0.249
Gender	Male	3001	34 (65.4)	2967 (70.9)	0.383
	Female	1235	18 (34.6)	1217 (29.1)	
Body mass index (kg/cm^2^)	Mean ± STD	24.54 ± 3.36	23.28 ± 3.32	24.55 ± 3.36	0.009
Diabetes	Yes	594	6 (11.5)	588 (14.1)	0.602
Hypertension	Yes	1592	16 (30.8)	1576 (37.7)	0.294
Chronic renal failure	Yes	86	1 (1.9)	85 (2.0)	> .999
ECOG	0,1,2	3410	42 (80.8)	3368 (80.5)	> .999
	3,4	57	0 (0.0)	57 (1.4)	
ASA	1	1417	11 (21.2)	1406 (33.6)	0.376
	2	1716	22 (42.3)	1694 (40.5)	
	3	175	2 (3.9)	173 (4.1)	
	4	5	0 (0.0)	5 (0.1)	
Hb	Median (IQR)	13.9 (12.7–15)	12.8 (11.2–13.9)	13.9 (12.7–15.05)	< .001
Platelet	Median (IQR)	229 (193–271)	279.5 (216–321)	229 (192–270)	< .001
Creatinine	Median (IQR)	0.99 (0.81–1.1)	0.95 (0.86–1.14)	0.99 (0.81–1.1)	0.807
Albumin	Median (IQR)	4.4 (4.1–4.6)	4.1 (3.7–4.3)	4.4 (4.1–4.6)	< .001
AST	Median (IQR)	21 (18–28)	20 (14–25)	21 (18–28)	0.041
ALT	Median (IQR)	21 (15–32)	16 (12–26)	21 (15–32)	0.004
MDRD GFR	Median (IQR)	75.5 (63.9–87.8)	71.95 (59.55–86.1)	75.5 (63.9–87.8)	0.245
Tumor location	Lt	2101	24 (46.2)	2077 (49.6)	0.687
	Rt	2056	28 (53.9)	2028 (48.5)	
	Bilat	27	0 (0.0)	27 (0.7)	
Nephrectomy	Open surgery	1930	38 (73.1)	1892 (45.2)	< .001
	Laparoscopic	2254	14 (26.9)	2240 (53.5)	
Operative extent	Partial	1851	4 (7.7)	1847 (44.1)	< .001
	Radical	1382	28 (53.9)	1354 (32.4)	
pT	T1	3393	14 (26.9)	3379 (80.8)	< .001
	T2	334	11 (21.2)	323 (7.7)	
	T3	486	26 (50.0)	460 (11.0)	
	T4 + Tx	23	1 (1.9)	22 (0.5)	
Histology	Clear cell	3575	32 (61.5)	3543 (84.7)	< .001
	Non-clear cell	602	17 (32.7)	585 (14.0)	
	Mixed	45	1 (1.9)	44 (1.1)	
Nuclear grade	Grade 1–2	2195	13 (25)	2182 (52.2)	0.003
	Grade 3–4	1832	28 (53.9)	1804 (43.1)	
Sarcomatoid differentiation	Yes	78	5 (9.6)	73 (1.7)	0.003
Necrosis	Yes	311	16 (30.8)	295 (7.1)	< .001
Lymphovascular invasion	Yes	159	18 (34.6)	141 (3.4)	< .001
Capsular invasion	Yes	718	16 (30.8)	702 (16.8)	0.008

### Multivariate analyses of risk factors for survival

Firstly, multivariable analysis defining any significant risk factors for each survival measures was performed (Tables 2, 3). The significantly common risk factors of all survival measures were Furhmann nuclear grade 3–4 and pathological TN stage with unfavorable hazard ratios (*p* < 0.05). As for the pT and pN stage, the hazard ratio of pT1-4N1 group indicated similar to or significantly greater than pT3N0 (HR 6.53 vs HR 6.53 for MFS; HR 6.67 vs HR 3.66 for RFS; HR 5.86 vs HR 2.14 for OS; and HR 9.33 vs HR 4.14 for CSS) (*p* < 0.05, Tables 2, 3). Other significant clinicopathological factors were summarized in Table 2 for MFS and RFS and in Table 3 for OS and CSS. As for the favorable risk factors with hazard ratio less than 1.0, non-clear cell histology was significant for both MFS and RFS, body mass index, preoperative hemoglobin level, and laparoscopic nephrectomy type for RFS, OS and CSS (*p* < 0.05).

**Table 2:** Univariable and multivariable Cox proportional hazard model of metastasis-free survival (MFS) and recurrence-free survival (RFS) in five pTN groups.

		MFS (n = 4236, event = 141)				RFS (n = 4236, event = 410)			
		Univariable model		Multivariable model		Univariable model		Multivariable model	
		HR (95% CI)	*p* value	HR (95% CI)	*p* value	HR (95% CI)	*p* value	HR (95% CI)	*p* value
Group	pT1N0	1 (ref)		1 (ref)		1 (ref)		1 (ref)	
	pTxN1	6.15 (2.22–17.04)	0.001	6.53 (2.3–18.56)	< .001	15.71 (10.45–23.6)	< .001	6.67 (4.06–10.94)	< .001
	pT2N0	5.34 (3.38–8.45)	< .001	5.14 (3.22–8.21)	< .001	4.51 (3.42–5.94)	< .001	2.18 (1.61–2.96)	< .001
	pT3N0	8.44 (5.73–12.44)	< .001	6.53 (4.37–9.76)	< .001	6.92 (5.50–8.70)	< .001	3.66 (2.81–4.76)	< .001
	pT4N0	11.75 (3.66–37.75)	< .001	7.08 (2.18–22.99)	0.001	17.76 (10.09–31.25)	< .001	5.26 (2.79–9.88)	< .001
Age at operation		1.02 (1.01–1.04)	0.003			1.01 (1.00–1.02)	0.005		
Body mass index (kg/cm^2^)		0.97 (0.92–1.02)	0.255			0.94 (0.91–0.97)	< .001	0.96 (0.93–0.99)	0.022
Diabetes	Yes	1.06 (0.66–1.72)	0.806			1.13 (0.86–1.49)	0.380		
Hypertension	Yes	1.65 (1.18–2.31)	0.004	1.50 (1.06–2.12)	0.021	1.10 (0.90–1.34)	0.380		
ASA	1 + 2	1 (ref)				1 (ref)		1 (ref)	
	3 + 4	1.20 (0.53–2.73)	0.667			2.52 (1.71–3.70)	< .001	2.21 (1.49–3.29)	< .001
Hb	Female (≤ 12), male (≤ 13)	1 (ref)				1 (ref)		1 (ref)	
	Female (> 12), male (> 13)	0.49 (0.34–0.70)	< .001			0.39 (0.32–0.48)	< .001	0.76 (0.60–0.96)	0.020
Platelet	≥ 150, ≤ 450	1 (ref)		1 (ref)		1 (ref)		1 (ref)	
	< 150	0.90 (0.42–1.93)	0.780	0.96 (0.45–2.06)	0.909	0.43 (0.22–0.83)	0.012	0.38 (0.19–0.77)	0.007
	> 450	5.25 (2.44–11.3)	< .001	2.18 (1.00–4.75)	0.051	5.17 (3.21–8.33)	< .001	1.77 (1.07–2.92)	0.027
Creatinine	≤ 1.3	1 (ref)				1 (ref)			
	> 1.3	1.49 (0.87–2.54)	0.150			1.43 (1.03–1.98)	0.034		
Albumin	≤ 3.0	1 (ref)				1 (ref)			
	> 3.0	0.61 (0.19–1.90)	0.391			0.47 (0.26–0.85)	0.013		
Nephrectomy	Open surgery	1 (ref)				1 (ref)		1 (ref)	
	Laparoscopic	0.55 (0.38–0.79)	0.001			0.35 (0.28–0.44)	< .001	0.60 (0.46–0.77)	< .001
Operative extent	Partial	1 (ref)				1 (ref)		1 (ref)	
	Radical	3.30 (1.91–5.70)	< .001			3.72 (2.72–5.09)	< .001	1.83 (1.28–2.61)	0.001
Histology	Clear cell	1 (ref)		1 (ref)		1 (ref)		1 (ref)	
	Non-clear cell	0.64 (0.35–1.15)	0.134	0.45 (0.25–0.82)	0.009	0.69 (0.50–0.97)	0.030	0.52 (0.37–0.74)	< .001
	Mixed	1.74 (0.43–7.03)	0.440	1.77 (0.43–7.28)	0.426	1.70 (0.76–3.81)	0.198	1.07 (0.46–2.51)	0.875
Nuclear grade	Grade 1–2	1 (ref)		1 (ref)		1 (ref)		1 (ref)	
	Grade 3–4	4.06 (2.72–6.06)	< .001	2.62 (1.73–3.98)	< .001	2.98 (2.35–3.78)	< .001	1.95 (1.50–2.53)	< .001
Sarcomatoid differentiation	Yes	6.37 (3.34–12.16)	< .001			4.60 (2.96–7.14)	< .001	1.72 (1.04–2.86)	0.036
Necrosis	Yes	3.85 (2.57–5.77)	< .001			2.72 (2.08–3.54)	< .001	1.44 (1.02–2.02)	0.039
Lymphovascular invasion	Yes	3.51 (2.11–5.83)	< .001			3.67 (2.70–4.99)	< .001		
Capsular invasion	Yes	1.74 (1.20–2.51)	0.004			1.40 (1.12–1.77)	0.004

**Table 3:** Univariable and multivariable Cox proportional hazard model of overall survival (OS) and cancer-specific survival (CSS) in five pTN groups.

		OS (n = 4236, event = 351)				CSS (n = 4236, event = 212)			
		Univariable model		Multivariable model		Univariable model		Multivariable model	
		HR (95% CI)	*p* value	HR (95% CI)	*p* value	HR (95% CI)	*p* value	HR (95% CI)	*p* value
Group	pT1N0	1 (ref)		1 (ref)		1 (ref)		1 (ref)	
	pTxN1	8.78 (5.58–13.83)	< .001	5.86 (3.41–10.06)	< .001	15.22 (8.87–26.12)	< .001	9.34 (5.01–17.41)	< .001
	pT2N0	1.77 (1.26–2.49)	0.001	1.15 (0.80–1.66)	0.457	3.24 (2.13–4.94)	< .001	2.13 (1.35–3.36)	0.001
	pT3N0	4.13 (3.23–5.29)	< .001	2.14 (1.61–2.83)	< .001	7.53 (5.48–10.34)	< .001	4.12 (2.90–5.87)	< .001
	pT4N0	11.46 (6.05–21.69)	< .001	4.89 (2.39–9.99)	< .001	25.24 (13.04–48.86)	< .001	10.33 (4.93–21.63)	< .001
Age at operation		1.05 (1.04–1.06)	< .001	1.03 (1.02–1.04)	< .001	1.03 (1.02–1.04)	< .001	1.02 (1.00–1.03)	0.032
Body mass index (kg/cm^2^)		0.92 (0.88–0.95)	< .001	0.93 (0.89–0.96)	< .001	0.90 (0.86–0.94)	< .001	0.91 (0.86–0.95)	< .001
Diabetes	Yes	1.89 (1.46–2.45)	< .001	1.75 (1.31–2.33)	< .001	1.82 (1.31–2.54)	< .001	2.21 (1.53–3.18)	< .001
Hypertension	Yes	1.43 (1.15–1.78)	0.001	1.35 (1.04–1.75)	0.023	1.32 (0.99–1.74)	0.055		
ASA	1 + 2	1 (ref)		1 (ref)		1 (ref)		1 (ref)	
	3 + 4	4.23 (3.01–5.94)	< .001	3.08 (2.15–4.41)	< .001	3.27 (2.01–5.31)	< .001	2.37 (1.41–3.96)	0.001
Hb	Female (≤ 12), male (≤ 13)	1 (ref)		1 (ref)		1 (ref)		1 (ref)	
	Female (> 12), male (> 13)	0.27 (0.21–0.34)	< .001	0.50 (0.39–0.64)	< .001	0.25 (0.19–0.33)	< .001	0.53 (0.39–0.74)	< .001
Platelet	≥ 150, ≤ 450	1 (ref)				1 (ref)			
	< 150	1.33 (0.82–2.15)	0.253			0.67 (0.30–1.53)	0.343		
	> 450	3.61 (1.91–6.82)	< .001			3.85 (1.80–8.24)	0.001		
Creatinine	≤ 1.3	1 (ref)				1 (ref)			
	> 1.3	2.44 (1.81–3.31)	< .001			2.56 (1.75–3.76)	< .001		
Albumin	≤ 3.0	1 (ref)				1 (ref)			
	> 3.0	0.27 (0.15–0.47)	< .001			0.22 (0.12–0.42)	< .001		
Nephrectomy	Open surgery	1 (ref)		1 (ref)		1 (ref)		1 (ref)	
	Laparoscopic	0.32 (0.24–0.41)	< .001	0.61 (0.45–0.83)	0.002	0.30 (0.21–0.42)	< .001	0.63 (0.43–0.93)	0.019
Operative extent	Partial	1 (ref)		1 (ref)		1 (ref)			
	Radical	2.76 (1.95–3.91)	< .001	1.51 (1.03–2.22)	0.036	4.18 (2.51–6.97)	< .001		
Histology	Clear cell	1 (ref)		1 (ref)		1 (ref)		1 (ref)	
	Non-clear cell	0.91 (0.65–1.28)	0.579	0.89 (0.62–1.28)	0.535	0.99 (0.65–1.51)	0.976	0.94 (0.60–1.46)	0.782
	Mixed	3.32 (1.64–6.70)	0.001	2.21 (0.99–4.94)	0.054	4.66 (2.19–9.95)	< .001	2.93 (1.25–6.90)	0.014
Nuclear grade	Grade 1–2	1 (ref)		1 (ref)		1 (ref)		1 (ref)	
	Grade 3–4	2.32 (1.78–3.04)	< .001	1.73 (1.29–2.32)	< .001	3.83 (2.64–5.56)	< .001	2.68 (1.78–4.04)	< .001
Sarcomatoid differentiation	Yes	3.85 (2.25–6.58)	< .001	1.98 (1.09–3.60)	0.026	5.73 (3.26–10.07)	< .001	1.85 (0.97–3.52)	0.063
Necrosis	Yes	1.80 (1.30–2.51)	0.001			2.70 (1.88–3.89)	< .001		
Lymphovascular invasion	Yes	2.64 (1.85–3.76)	< .001			3.56 (2.37–5.34)	< .001		
Capsular invasion	Yes	1.02 (0.78–1.33)	0.877			1.25 (0.90–1.73)	0.185		

Several significant factors such as hypertension (HR 1.50) were detected for MFS; ASA score 3–4 (HR 2.21), thrombocytosis (> 45,000/dL, HR 1.77), radical operative extent (HR 1.83), sarcomatoid differentiation (HR 1.72), and tumor necrosis (HR 1.44) were found for RFS (*p* < 0.05, Table 2); age (HR 1.03), diabetes (HR 1.75), hypertension (HR 1.35), ASA score 3–4 (HR 3.08), radical operative extent (HR 1.51), and presence of sarcomatoid differentiation (HR 1.98) were identified for OS; and age (HR 1.02), diabetes (HR 2.21), ASA score 3–4 (HR 2.37), and mixed cell histology (HR 2.93) were for detected CSS (*p* < 0.05, Table 3).

### Comparison of survival outcomes according to pathologic stages

Further analyses comparing survival outcomes of four groups according to pT and pN stagings are shown after adjusted by the significant clinicopathological parameters for each survival measures among 4236 patients in Table 4 and Supplementary Table 2. The HR of pT1-2N1 group were significantly higher than that of T3-4N0 group and lower than that of pT3-4N1 in all the MFS/RFS/OS/CSS, respectively (*p* < 0.05, Table 4A). A subgroup analysis was also performed only with 1382 radical nephrectomized cases to compare the survival outcomes according to the pT and pN stagings (Table 4B). The results of this subgroup analysis were similar those of the analysis involving all patients: the HR of the pT1-2N1 group were significantly higher than that of the T3-4N0 group in all the MFS/RFS/OS/CSS, but it was significantly lower than (HR 2.51) that of the pT3-4N1 group in all the survival outcomes, except for the RFS (HR 2.87), respectively (*p* < 0.05).

**Table 4:** Cox proportional hazard model of MFS, RFS, OS, and CSS in four pTN groups (A) among overall 4236 patients and (B) among only 1382 radical nephrectomized cases.

Group	MFS (n = 4236, event = 141)						RFS (n = 4236, event = 410)					
	N	Event (%)	Univariable model		Multivariable model		N	Event (%)	Univariable model		Multivariable model	
			HR (95% CI)	*p* value	HR (95% CI)	*p* value			HR (95% CI)	*p* value	HR (95% CI)	*p* value
**(A)**												
pT3-4N0	482	57 (11.8)	1 (ref)		1 (ref)		482	144 (29.9)	1 (ref)		1 (ref)	
pT1-2N0	3702	80 (2.2)	0.17 (0.12–0.24)	< .001	0.32 (0.23–0.47)	< .001	3702	239 (6.5)	0.18 (0.15–0.22)	< .001	0.34 (0.27–0.43)	< .001
pT1-2N1	25	3 (12.0)	1.06 (0.33–3.39)	0.922	1.34 (0.41–4.33)	0.629	25	10 (40.0)	1.38 (0.73–2.63)	0.322	1.09 (0.51–2.35)	0.820
pT3-4N1	27	1 (3.7)	0.38 (0.05–2.72)	0.332	0.42 (0.06–3.09)	0.394	27	17 (63.0)	3.21 (1.94–5.30)	< .001	2.75 (1.53–4.96)	0.001

Group	OS (n = 4236, event = 351)						CSS (n = 4236, event = 212)					
	N	Event (%)	Univariable model		Multivariable model		N	Event (%)	Univariable model		Multivariable model	
			HR (95% CI)	*p* value	HR (95% CI)	*p* value			HR (95% CI)	*p* value	HR (95% CI)	*p* value
pT3-4N0	482	109 (22.6)	1 (ref)		1 (ref)		482	87 (18.1)	1 (ref)		1 (ref)	
pT1-2N0	3702	221 (6.0)	0.25 (0.20–0.32)	< .001	0.48 (0.37–0.62)	< .001	3702	109 (2.9)	0.15 (0.12–0.20)	< .001	0.27 (0.20–0.37)	< .001
pT1-2N1	25	10 (40.0)	1.81 (0.95–3.46)	0.074	2.11 (1.05–4.26)	0.036	25	8 (32.0)	1.76 (0.85–3.64)	0.128	1.93 (0.83–4.51)	0.127
pT3-4N1	27	11 (40.7)	2.27 (1.22–4.22)	0.010	3.49 (1.73–7.05)	0.001	27	8 (29.6)	2.02 (0.98–4.17)	0.058	2.73 (1.26–5.93)	0.011

Group	MFS (n = 1382, event = 56)						RFS (n = 1382, event = 173)					
	N	Event (%)	Univariable model		Multivariable model		N	Event (%)	Univariable model		Multivariable model	
			HR (95% CI)	*p* valve	HR (95% CI)	*p* valve			HR (95% CI)	*p* valve	HR (95% CI)	*p* value
**(B)**												
pT3-4N0	246	27 (11.0)	1 (ref)		1 (ref)		246	62 (25.2)	1 (ref)		1 (ref)	
pT1-2N0	1108	26 (2.4)	0.18 (0.11–0.32)	< .001	0.31 (0.18–0.56)	< .001	1108	97 (8.8)	0.27 (0.20–0.38)	< .001	0.54 (0.36–0.79)	0.002
pT1-2N1	14	3 (21.4)	2.33 (0.75–7.23)	0.145	2.16 (0.69–6.77)	0.188	14	6 (42.9)	1.92 (0.83–4.44)	0.128	1.35 (0.57–3.18)	0.499
pT3-4N1	14	0 (0.0)	0.33 (0.02–5.78)	0.451	0.23 (0.01–4.10)	0.317	14	8 (57.1)	3.31 (1.58–6.91)	0.002	1.54 (0.70–3.36)	0.280

Group	OS (n = 1382, event = 140)						CSS (n = 1382, event = 84)					
	N	Event (%)	Univariable model		Multivariable model		N	Event (%)	Univariable model		Multivariable model	
			HR (95% CI)	*p* value	HR (95% CI)	*p* value			HR (95% CI)	*p* value	HR (95% CI)	*p* value
pT3-4N0	246	42 (17.1)	1 (ref)		1 (ref)		246	32 (13.0)	1 (ref)		1 (ref)	
pT1-2N0	1108	88 (7.9)	0.36 (0.25–0.52)	< .001	0.64 (0.43–0.95)	0.028	1108	44 (4.0)	0.24 (0.15–0.38)	< .001	0.52 (0.31–0.87)	0.013
pT1-2N1	14	5 (35.7)	1.93 (0.76–4.88)	0.167	4.51 (1.57–12.98)	0.005	14	4 (28.6)	1.99 (0.70–5.64)	0.196	3.00 (0.87–10.30)	0.081
pT3-4N1	14	5 (35.7)	1.95 (0.77–4.94)	0.160	3.21 (1.1–9.41)	0.034	14	4 (28.6)	2.03 (0.72–5.75)	0.184	2.73 (0.92–8.08)	0.070

(Panel A) Adjusted for hypertension, operative extent, histology, nuclear grade, and necrosis in multivariable model of MFS. Adjusted for BMI, ASA, hemoglobin, platelet, nephrectomy, operative extent, histology, nuclear grade, and necrosis in multivariable model of RFS. Adjusted for age, BMI, diabetes, hypertension, ASA, hemoglobin, nephrectomy, operative extent, nuclear grade, and sarcomatoid differentiation in multivariable model of OS. Adjusted for BMI, diabetes, hypertension, ASA, hemoglobin, nephrectomy, histology, and nuclear grade in multivariable model of CSS.

(Panel B) Adjusted for nuclear grade and necrosis in multivariable model of MFS. Adjusted for ASA, platelet, nephrectomy, nuclear grade, sarcomatoid differentiation, and necrosis in multivariable model of RFS. Adjusted for age, BMI, diabetes, ASA, hemoglobin, nuclear grade, and sarcomatoid differentiation in multivariable model of OS. Adjusted for age, BMI, hemoglobin, creatinine, nuclear grade, sarcomatoid differentiation, and necrosis in multivariable model of CSS.

**Figure 1:**
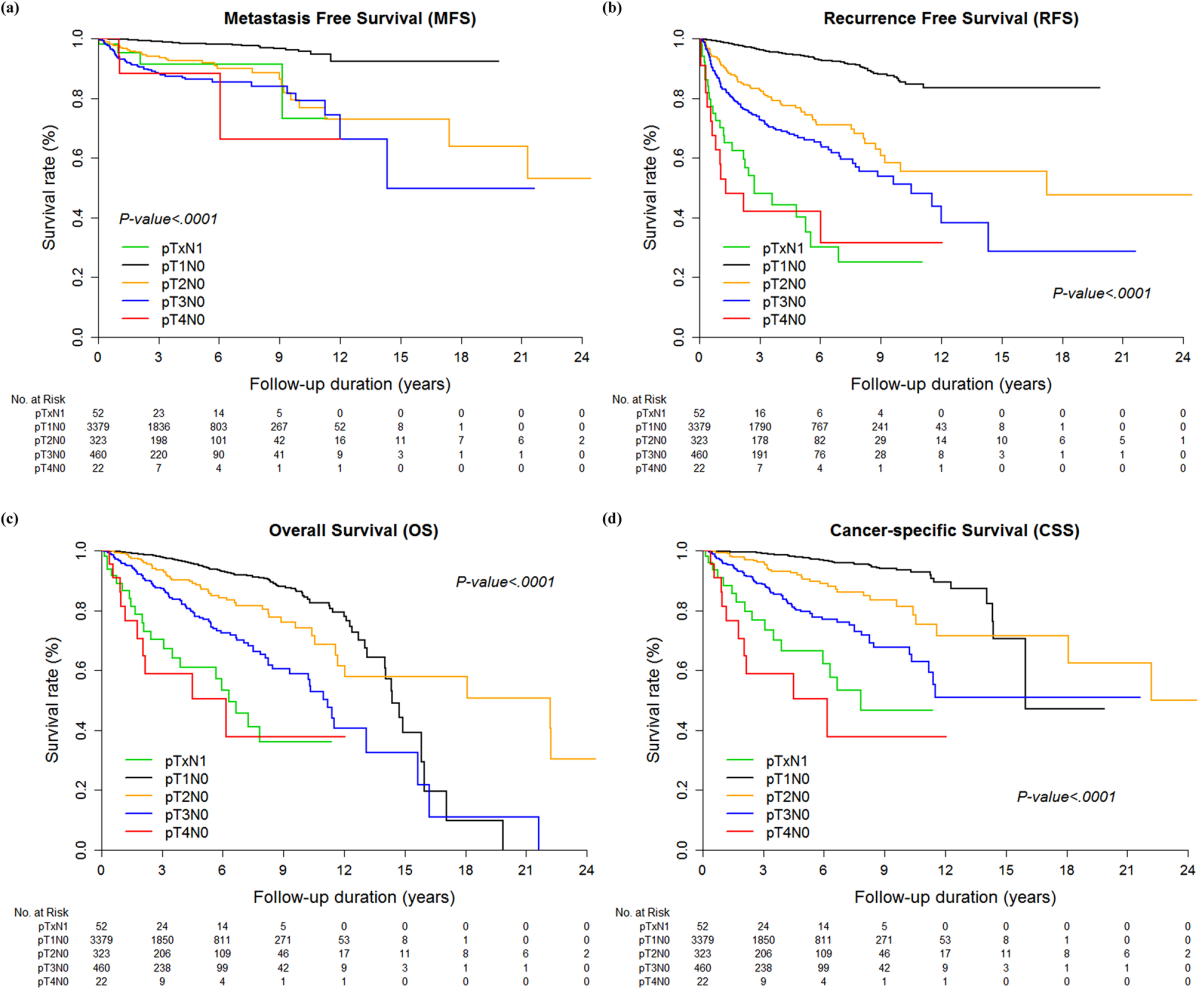
Kaplan–Meier curves for (**a**) MFS, (**b**) RFS, (**c**) OS, and (**d**) CSS in five pTN groups.

**Figure 2:**
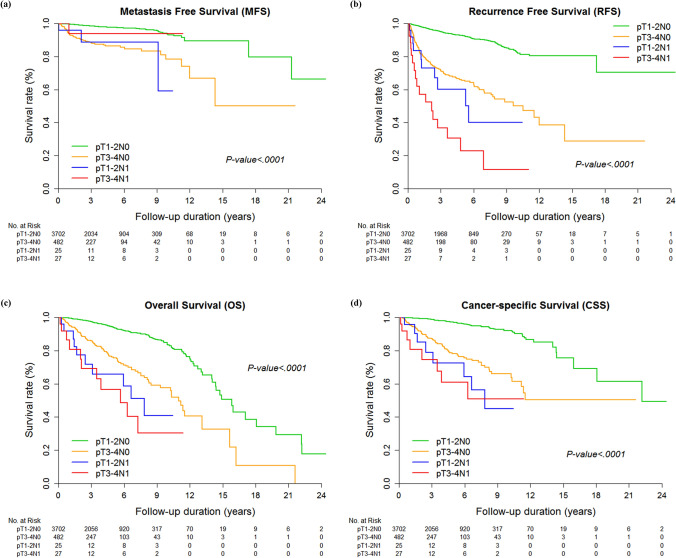
Kaplan–Meier curves for (**a**) MFS, (**b**) RFS, (**c**) OS, and (**d**) CSS in four pTN groups.

Kaplan–Meier curves for RFS, MFS, OS, and CSS in relation to pT and pN stage confirmed that the prognostic survivals were significantly different among stratified pTN stage groups among all the enrolled patients (*p* < 0.001, Figs. 1, 2). In subgroup analysis with only nodal positive patients, there was no statistically significant difference according to tumor histology or pathologic T stage (*p* > 0.05, Supplementary Figs. 1, 2), except for RFS (*p* = 0.044, Supplementary Fig. 2B).

### Comparison of survival outcomes according to pathologic nodal positivity only among LN-dissected patients

Additional comparative sub-analyses for each survival outcome were performed according to pathologic nodal states only among patients who underwent nodal dissection (Supplementary Tables 3 and 4). The baseline comparison showed that body mass index, preoperative hemoglobin/platelet/albumin/ALT, operative extent, pT staging, histology, nuclear grade, sarcomatoid differentiation, necrosis, lymphovascular invasion, and capsular invasion were significantly different between group pN1 and group pN0 (Supplementary Table 3). The multivariate survival analyses showed that the pN1 group had significantly worse RFS (HR 3.37, CI 2.06–5.53), OS (HR 3.49, CI 2.05–5.93), and CSS (HR 3.6, CI 1.72–6.20) than the pN0 group (*p* < 0.05), whereas the MFS was insignificantly different between both groups (*p* = 0.2351) (Supplementary Table 4).

### Comparison of survival outcomes according to nodal status and LN dissection in pT2-4 staged patients

Of 843 patients staged pT2-4, 335 did not undergo LN dissection, whereas the remaining patients underwent LN dissection. The baseline characteristics (Supplementary Table 5) and survival outcomes (Supplementary Table 6) were compared between LN dissected (pN0 [N = 470] and pN1 [N = 38]) and LN non-dissected patients (pNx [N = 335]). Baseline comparison showed significant differences among the three groups (p < 0.05, Supplementary Table 5). The comparison between the LND and non LND groups showed that the LND group had significantly worse MFS (HR 2.33, CI 1.42–3.82), OS (HR 1.81, CI 1.11–2.94), and CSS (HR 1.79, CI 1.03–3.10) than the non LND group (*p* < 0.05, Supplementary Table 6). Regarding their survival comparison, the pN0 group had significantly worse MFS (HR 2.36, CI 1.43–3.91) than the other two groups, whereas the pN1 had significantly worse RFS (HR 2.69, CI 1.48–4.89), OS (HR 4.44, CI 2.24–8.82), and CSS (HR 4.38, CI 2.08–9.26) than the pNx group (as a reference group, HR = 1.0) (*p* < 0.05, Supplementary Table 6).

## Discussion

The prognostic efficacy of LN dissection^12^ and the LN+ after either radical or partial nephrectomy in nmRCC, especially whether to apply adjuvant systemic therapy or not for better survival outcomes and to decrease the local recurrence and distant metastasis in spite of several failed clinical trials to demonstrate its efficacy on survival measures^9,13^, have always the main debating issues for clinicians. Various concepts of indications for LN dissection in nmRCC have evolved as a necessary intraoperative procedure for the specific cohort with LN positivity or high risk factors^7–9,11,19^, because LN+ provides useful information for postoperative therapeutic management^2,3,5,11^ and has always been a significant unfavorable risk factor for survival. This study also supported the unfavorable outcome of the LN+ group with even low T1-2 stage, and the LN+ group had worse HRs than the T3-4N0 group after adjusting for the significant clinicopathological factors for each survival measure (Table 4 and Suppl. Table 2). This indicates that the low tumor burden in the localized primary kidney tumor in the LN+ group should not be considered as a localized RCC, but as a T3-4N0 of high tumor burden, because the cancer cells have already disrupted the lymphatic drainage system to spread to the locoregional area. Therefore, the microscopically locoregional advanced low T staged LN+ group might be treated surgically with LN dissection, and any attempts to identify the aggressive or unfavorable characteristics of localized primary RCC with even low T1-2 stage should be evaluated preoperatively and thoroughly with improved imaging studies to identify the presence of any high-infiltrating risk factor of primary RCC into the LN. In addition, this study evaluated the prognostic impact of LND on each survival outcome compared to non-LND, especially for pT2-4 staged patients (Supplementary Table 6). The comparison of survival outcomes showed that patients who underwent LN dissection for the suspected LN positivity either preoperatively or intraoperatively had worse survival outcomes than the non-LND group. Even patients staged pN0 in the LND group had significantly worse MFS than the non-LND group (designated as pNx), indicating the necessity of indications for LN dissection during radical/partial nephrectomy in RCC.

A recent study from Raddia et al. supported our findings, and they suggested that the thorough clinical staging with preoperative imaging studies was an important factor for identifying the high-risk LN+ group and providing the beneficiary role of LND in the survival outcomes of nmRCC patients who underwent curative nephrectomy^20,21^. In these backgrounds, a recent adjuvant clinical trial suggesting several eligibility and radiologic assessments for kidney cancer that either CT or MRI within 4 weeks should be performed, and every patient with microscopically positive soft tissue or vascular margins without gross residual or radiologic disease may be included in trials to all suspicious regional lymph nodes^22^. Thus, previous papers supported the complete removal of suspicious LN, and the efficacy of LN dissection is important for survival prognoses^22–24^. Babaian et al.^23^ showed the effectiveness of LN dissection in 1270 nmRCC patients and reported that OS, CSS, and RFS among pNx, pN0, and pN1 cases were statistically significant (*p* < 0.001). In addition, similarities were observed in the results of the additional sub-analyses regarding the comparison of each survival outcome (Table 4), even among patients who underwent only LN dissection (Supplementary Table 4). The LN+ group had significantly worse outcomes in terms of RFS, OS, and CSS than the LN− group (*p* < 0.05, Table 4, and Supplementary Table 4). Paparel et al.^24^ also showed in their study of 72 patients with local recurrence after radical nephrectomy that the completeness of the surgical treatment of local recurrence was a major significant prognostic factor for survival. This study showed some potential high risk factors of postoperative disease recurrences. The baseline factors, such as high ASA score, hypertension, and thrombocytosis, might be high risk factors for both LN dissection during nephrectomy and disease recurrence. Other pathologic factors, such as nuclear grade, histology, sarcomatoid differentiation, and tumor necrosis, might also provide some useful information about the potential risk of disease recurrence with or without LN dissection.

Another important issue to discuss from the findings of this study was about the differences in the significant risk factors of each survival outcome. There were differences in the significant risk factors between RFS and MFS and also similarities in the significant risk factors among RFS, OS, and CSS (Tables 2, 4), referring to the fact that the LN+ more affected local recurrence leading to OS and CSS than distant metastasis (Supplementary Table 1). These similarities in the significant risk factors between OS and CSS and the difference in the risk factors between MFS and OS as well as CSS were also observed in the study by Gershman et al.^25^. In their study, the risk factors of OS and CSS were tumor necrosis, sarcomatoid component, and baseline ECOG, whereas those of MFS were symptom presentation, IVC thrombi, histology, and pT4.

The LN+ results from the loco-regionally disrupted lymphatic spread of cancer cells from the primary kidney tumor, whereas distant metastasis results from the hematogenous dissemination of cancer cells, which required time for settlement of cancer cells after curative nephrectomy in nmRCC^2, 26,27^. This was the reason why the risk factors were significantly different between RFS and MFS, especially when identifying the significant factors of each survival measure among 52 LN+ patients (Supplementary Table 1). No factors were found to be significant in MFS, whereas the preoperative clinical factors relating to performance status such as age, diabetes, and preoperative hemoglobin level were found to be significant in RFS, similar to those of OS and CSS (*p* < 0.05). Therefore, the LN+ more affects the lymphatic local recurrence than distant metastasis, which significantly influences OS and CSS, because postoperative distant metastasis occurs via blood vessels, which requires a sufficient time to allow cancer cells to settle down at a new distant organ lesion after overcoming the immune system. Contrarily, the lymphatic spread in local recurrence at the time of nephrectomy easily occurred owing to the disrupted lymphatic nodal system, and it has a greater influence on OS and CSS than distant metastasis after surgery^26–28^.

Furthermore, regarding postoperative follow-up regimen, this study provides some clues about the postoperative surveillance for LN+ patients equivalent to those with locally advanced RCC (pT3-4N0) (Fig. 1a). As mentioned previously, the settlement time for metastatic cells detected on follow-up imaging was longer than that of locally recurred cancer cells, indicating that metastasis slowly progressed even after a certain time has passed since the nephrectomy. Therefore, it would be unnecessary to overutilize postoperative metastatic imaging work-ups for the low T staged LN− group after curative either radical or partial nephrectomy, whereas the LN+ group and those patients with high risk factors, such as nuclear grade, histology, sarcomatoid differentiation, and tumor necrosis, should be evaluated thoroughly with postoperative imaging studies for locoregional recurrence, as well as metastatic work-ups after a certain time has passed since the safety time from the local recurrence. Thus, additional work will be needed to assess the impact of the guidelines on the differential management of LN+ from that of LN-group RCC, taking into consideration that local recurrence might occur before distant metastasis; A thorough examination on local recurrence should be more focused on the postoperative follow-up for the LN+ and T3N0 groups^4^.

As for the adjuvant therapeutic modalities and their postoperative application timing in the LN+ group, the present study showed that the LN+ group had higher probability of locoregional recurrence affecting CSS and OS survival, rather than distant metastasis. The locoregional recurrence led to cancer death by invasion to adjacent organs, such as the bowel, pancreas, and hepatobiliary system as well as distant metastasis seeded through the circulatory system. Considering this, a combination of adjuvant local surgery, such as bowel resection or retroperitoneal LN dissection, and a systemic immune checkpoint inhibitor might also be an option for patients with locoregionally isolated recurrent lesions or those at high risk of tumor invasion to adjacent organs after curative nephrectomy. Itano et al. investigated 30 patients with isolated local fossa carcinoma recurrence after complete radical nephrectomy without evidence of metastatic disease and reported that patients who underwent additional surgical resection had an improved 5-year CSS rate of 51% compared to the CSS rate of 18% in patients who underwent adjuvant medical therapy and of 13% in those who were managed by observation alone^29^. Barbian et al.^23^ also stated the importance of complete retroperitoneal LN dissection in favor of CSS. In this approach, the immune checkpoint inhibitor works systemically to induce immune enhancement, whereas local surgery relieves the direct invasion. Several adjuvant trials of combined surgery and systemic therapies are ongoing for the primary objective of survival measures, as well as cancer-relating symptom-free survivals^30^.

The neoadjuvant combination therapy might also be a new good option for the clinical LN+ group. Given that the LN+ at preoperative imaging provides data on the therapeutic responsiveness of the targeted lesion, these patients might be a good indication for the neoadjuvant targeted therapy before nephrectomy for successful tumor reduction, and a number of phase II studies and a recently published small randomized double-blind placebo-controlled study showed the favorable outcome of neoadjuvant therapies^30^. As for the adjuvant combination therapy, targeted agents combined with immunomodulatory immune check-point inhibitor after nephrectomy to enhance the surgical outcome in multiple survival measures might also be another new option, because targeted agents, with their acute onset, may be able to compensate for the time necessary for the delayed initiation of immune reaction after antigen presentation and for boosting the immune system. The JAVELIN Renal 101 Clinical Trial for locally advanced RCC trial of first-line combination therapy expected favorable outcomes in LN+ patients with significantly longer PFS with avelumab plus axitinib than with sunitinib^31^. If these ongoing trials have suggested the benefit role of adjuvant combination therapy for the LN+ group, then the LN dissection might be recommended during nephrectomy for nmRCC. However, the adjuvant combined therapy in nmRCC after nephrectomy might be cautiously considered because of the increased rate of adverse events graded 3 or higher and the absence of efficacy assessment without obvious targeted lesions. Further studies are warranted to determine the optimal timing and dosing of the combination of adjuvant therapies as well as their efficacy in high-risk patients without any targeted tumor lesion in nmRCC.

This study had a number of limitations, including the retrospective multi-centric design with short-term median follow-up of less than 5 years, the lack of a standardized surgical protocol and standardized indications for lymph node dissection, the low rate of LN dissection (43.1%) for selected patients, non-consideration of LN extent, localization, and the number LN removed, presence of tumor thrombi, symptom presentation, and the different baseline characteristics between the LN+ and LN− groups^25,32,33^. In addition, racial consideration for RCC and its survival prognoses should be similarly considered because the study participants totally comprised the Asian population. The higher prevalence of clear-cell histology and more favorable outcome of RCC in localized and metastatic states in Asians should be further evaluated in future studies, with different National Cancer coverage and screening systems and various cultures of the patient supporting family system. However, the different prognostic position of pathologic nodal positivity between MFS and RFS and the similarities of risk factors for RFS, OS, and CSS in this nmRCC cohort provide important information about the postoperative follow-up protocols for patients with risk factors for recurrence and metastasis and aid in the understanding of the pathologic lymph nodal positivity in nmRCC after curative nephrectomy. Considering reducing the difference in baseline characteristics, future prospective research encompassing larger sample sizes should be designed to compare the survival outcomes of LN+ and LN− groups.

## Conclusion

This large nmRCC cohort study revealed that pathologic nodal positive had significantly different from and worse effects on survival outcomes than any non-nodal T staged RCC after curative nephrectomy. pT1-2N1 group had worse prognoses than pT3-4N0 group in all the survival outcomes. Especially, pathologic node positivity had a greater effect on local recurrence than on distant metastasis. Further prospective studies of nodal positivity should be planned to evaluate the prognostic effects of node positivity in nmRCC.

## Supplementary Information

Supplementary Information

## Data Availability

The datasets generated during and/or analysed during the current study are available from the corresponding author on reasonable request.
