# Supplementary material for: Prognostic significance of pathologic nodal positivity in non-metastatic patients with renal cell carcinoma who underwent radical or partial nephrectomy
Source: Sci Rep. 2021 Feb 4;11:3079. doi: 10.1038/s41598-021-82750-y (PMC7862313; doi:10.1038/s41598-021-82750-y)
Supplement: Supplementary file 1 — Supplementary Information [file 41598_2021_82750_MOESM1_ESM.docx]

**Prognostic significance of pathologic nodal positivity in non-metastatic patients with renal cell carcinoma who underwent radical or partial nephrectomy**

Sung Han Kim, Boram Park, Eu Chang Hwang, Sung-Hoo Hong, Chang Wook Jeong, Cheol Kwak, Seok Soo Byun, Jinsoo Chung

**Supplementary Table 1. Univariable Cox proportional hazard model for MFS, RFS, OS, and CSS in LN(+) patients (N=52)**

|  | | MFS (n=52, event=4) | | RFS (n=52, event=27) | | OS (n=52, event=21) | | CSS (n=52, event=16) | |
| --- | --- | --- | --- | --- | --- | --- | --- | --- | --- |
|  |  | Univariable model | | Univariable model | | Univariable model | | Univariable model | |
|  |  | HR (95% CI) | p-value | HR (95% CI) | p-value | HR (95% CI) | p-value | HR (95% CI) | p-value |
| Age at operation |  | 1.11 (0.99-1.25) | 0.072 | 1.00 (0.98-1.03) | 0.815 | 1.04 (1.00-1.07) | 0.051 | 1.04 (1.00-1.08) | 0.076 |
| Body mass index (kg/cm2) |  | 0.84 (0.59-1.21) | 0.360 | 0.96 (0.84-1.09) | 0.491 | 0.93 (0.80-1.08) | 0.346 | 0.90 (0.76-1.07) | 0.224 |
| Diabetes | yes | 5.94 (0.51-69.26) | 0.155 | 4.12 (1.32-12.89) | 0.015 | 1.37 (0.32-5.94) | 0.676 | 0.88 (0.12-6.74) | 0.904 |
| Hypertension | yes | 2.69 (0.36-19.89) | 0.332 | 0.88 (0.36-2.20) | 0.789 | 0.96 (0.32-2.87) | 0.935 | 0.94 (0.27-3.35) | 0.929 |
| ASA | 1+2 |  |  | 1 (ref) |  | 1 (ref) |  | 1 (ref) |  |
|  | 3+4 |  |  | 4.62 (1.01-21.12) | 0.049 | 1.94 (0.25-15.28) | 0.529 | 2.73 (0.33-22.28) | 0.350 |
| Hb | female (≤12), male (≤13) | 1 (ref) |  | 1 (ref) |  | 1 (ref) |  | 1 (ref) |  |
|  | female (>12), male (>13) | 0.30 (0.03-3.42) | 0.331 | 0.25 (0.10-0.60) | 0.002 | 0.18 (0.07-0.49) | 0.001 | 0.13 (0.04-0.42) | 0.001 |
| Platelet | ≥150, ≤450 |  |  | 1 (ref) |  |  |  |  |  |
|  | <150 |  |  | 2.59 (0.33-20.21) | 0.364 |  |  |  |  |
|  | >450 |  |  | 3.21 (0.89-11.57) | 0.074 |  |  |  |  |
| Creatinine | ≤1.3 |  |  | 1 (ref) |  | 1 (ref) |  | 1 (ref) |  |
|  | >1.3 |  |  | 2.68 (0.90-8.02) | 0.078 | 0.68 (0.09-5.14) | 0.711 | 0.83 (0.11-6.30) | 0.853 |
| Albumin | ≤3.0 |  |  | 1 (ref) |  | 1 (ref) |  | 1 (ref) |  |
|  | >3.0 |  |  | 0.12 (0.03-0.58) | 0.008 | 0.08 (0.01-0.80) | 0.032 | 0.06 (0.01-0.68) | 0.023 |
| Nephrectomy | Open surgery | 1 (ref) |  | 1 (ref) |  | 1 (ref) |  | 1 (ref) |  |
|  | Laparoscopic | 1.06 (0.11-10.31) | 0.958 | 0.74 (0.28-1.97) | 0.552 | 0.15 (0.02-1.10) | 0.062 | 0.19 (0.03-1.46) | 0.111 |
| Operative Extent | partial |  |  |  |  |  |  |  |  |
|  | radical |  |  |  |  |  |  |  |  |
| Histology | clear cell | 1 (ref) |  | 1 (ref) |  | 1 (ref) |  | 1 (ref) |  |
|  | non-clear cell | 4.79 (0.48-47.70) | 0.181 | 0.63 (0.25-1.62) | 0.341 | 0.68 (0.24-1.90) | 0.463 | 0.32 (0.07-1.45) | 0.141 |
|  | mixed |  |  | 1.35 (0.18-10.20) | 0.775 | 2.31 (0.3-18.14) | 0.425 | 2.67 (0.33-21.39) | 0.356 |
| Nuclear grade | grade 1-2 |  |  | 1 (ref) |  | 1 (ref) |  | 1 (ref) |  |
|  | grade 3-4 |  |  | 1.16 (0.41-3.28) | 0.787 | 3.38 (0.75-15.15) | 0.112 | 5.07 (0.64-40.15) | 0.124 |
| Sarcomatoid differentiation | yes |  |  | 0.98 (0.23-4.23) | 0.983 | 1.63 (0.36-7.37) | 0.527 | 2.23 (0.47-10.52) | 0.312 |
| Necrosis | yes | 0.65 (0.07-6.30) | 0.712 | 0.86 (0.36-2.04) | 0.736 | 0.60 (0.22-1.65) | 0.327 | 0.87 (0.30-2.50) | 0.791 |
| Lymphovascular invasion | yes | 2.49 (0.34-18.01) | 0.367 | 1.14 (0.51-2.56) | 0.750 | 0.50 (0.18-1.38) | 0.183 | 0.73 (0.25-2.13) | 0.564 |
| Capsular invasion | yes | 0.82 (0.09-8.01) | 0.867 | 1.03 (0.46-2.30) | 0.942 | 0.60 (0.23-1.57) | 0.301 | 0.70 (0.24-2.04) | 0.515 |

MFS: metastasis-free survival, RFS : recurrence-free survival, OS : overall survival, CSS : cancer-specific survival

**Supplementary Table 2 Cox proportional hazard model of MFS, RFS, OS, and CSS in four pTN groups (A) among overall 4236 patients and (B) among only 1382 radical nephrectomized cases**

**(A)**

| Group | MFS (n=4236, event=141) | | | | | | RFS (n=4236, event=410) | | | | | |
| --- | --- | --- | --- | --- | --- | --- | --- | --- | --- | --- | --- | --- |
|  | N | Event (%) | Univariable model | | Multivariable model | | N | Event (%) | Univariable model | | Multivariable model | |
|  |  |  | HR (95% CI) | p-value | HR (95% CI) | p-value |  |  | HR (95% CI) | p-value | HR (95% CI) | p-value |
| pT1-2N0 | 3702 | 80 (2.2) | 1 (ref) |  | 1 (ref) |  | 3702 | 239 (6.5) | 1 (ref) |  | 1 (ref) |  |
| pT3-4N0 | 482 | 57 (11.8) | 5.85 (4.16-8.22) | <.001 | 3.08 (2.14-4.45) | <.001 | 482 | 144 (29.9) | 5.55 (4.51-6.83) | <.001 | 2.92 (2.31-3.69) | <.001 |
| pT1-2N1 | 25 | 3 (12.0) | 6.20 (1.95-19.64) | 0.002 | 4.12 (1.27-13.34) | 0.018 | 25 | 10 (40.0) | 7.68 (4.08-14.46) | <.001 | 3.19 (1.49-6.86) | 0.003 |
| pT3-4N1 | 27 | 1 (3.7) | 2.20 (0.31-15.80) | 0.434 | 1.30 (0.18-9.51) | 0.799 | 27 | 17 (63.0) | 17.80 (10.87-29.14) | <.001 | 8.03 (4.46-14.46) | <.001 |
|  |  | | | | | |  | | | | | |
| Group | OS (n=4236, event=351) | | | | | | CSS (n=4236, event=212) | | | | | |
|  | N | Event (%) | Univariable model | | Multivariable model | | N | Event (%) | Univariable model | | Multivariable model | |
|  |  |  | HR (95% CI) | p-value | HR (95% CI) | p-value |  |  | HR (95% CI) | p-value | HR (95% CI) | p-value |
| pT1-2N0 | 3702 | 221 (6.0) | 1 (ref) |  | 1 (ref) |  | 3702 | 109 (2.9) | 1 (ref) |  | 1 (ref) |  |
| pT3-4N0 | 482 | 109 (22.6) | 3.98 (3.16-5.01) | <.001 | 2.09 (1.62-2.70) | <.001 | 482 | 87 (18.1) | 6.50 (4.90-8.62) | <.001 | 3.73 (2.73-5.10) | <.001 |
| pT1-2N1 | 25 | 10 (40.0) | 7.20 (3.82-13.59) | <.001 | 4.41 (2.21-8.81) | <.001 | 25 | 8 (32.0) | 11.43 (5.56-23.47) | <.001 | 7.22 (3.10-16.80) | <.001 |
| pT3-4N1 | 27 | 11 (40.7) | 9.03 (4.92-16.57) | <.001 | 7.29 (3.62-14.7) | <.001 | 27 | 8 (29.6) | 13.11 (6.39-26.92) | <.001 | 10.20 (4.74-21.97) | <.001 |

Adjusted for hypertension, operative extent, histology, nuclear grade, and necrosis in multivariable model of MFS

Adjusted for BMI, ASA, hemoglobin, platelet, nephrectomy, operative extent, histology, nuclear grade, and necrosis in multivariable model of RFS

Adjusted for age, BMI, diabetes, hypertension, ASA, hemoglobin, nephrectomy, operative extent, nuclear grade, and sarcomatoid differentiation in multivariable model of OS

Adjusted for BMI, diabetes, hypertension, ASA, hemoglobin, nephrectomy, histology, and nuclear grade in multivariable model of CSS

**(B)**

| Group | MFS (n=1,382, event=56) | | | | | | RFS (n=1,382, event=173) | | | | | |
| --- | --- | --- | --- | --- | --- | --- | --- | --- | --- | --- | --- | --- |
|  | N | Event (%) | Univariable model | | Multivariable model | | N | Event (%) | Univariable model | | Multivariable model | |
|  |  |  | HR (95% CI) | p-value | HR (95% CI) | p-value |  |  | HR (95% CI) | p-value | HR (95% CI) | p-value |
| pT1-2N0 | 1108 | 26 (2.4) | 1 (ref) |  | 1 (ref) |  | 1108 | 97 (8.8) | 1 (ref) |  | 1 (ref) |  |
| pT3-4N0 | 246 | 27 (11.0) | 5.43 (3.15-9.36) | <.001 | 3.19 (1.80-5.66) | <.001 | 246 | 62 (25.2) | 3.67 (2.66-5.05) | <.001 | 1.87 (1.27-2.75) | 0.002 |
| pT1-2N1 | 14 | 3 (21.4) | 12.63 (4.06-39.33) | <.001 | 6.88 (2.14-22.13) | 0.001 | 14 | 6 (42.9) | 7.03 (3.08-16.06) | <.001 | 2.51 (1.03-6.15) | 0.044 |
| pT3-4N1 | 14 | 0 (0.0) | 1.82 (0.11-31.42) | 0.681 | 0.74 (0.04-13.23) | 0.835 | 14 | 8 (57.1) | 12.11 (5.88-24.96) | <.001 | 2.87 (1.25-6.58) | 0.013 |
|  |  | | | | | |  | | | | | |
| Group | OS (n=1,382, event=140) | | | | | | CSS (n=1,382, event=84) | | | | | |
|  | N | Event (%) | Univariable model | | Multivariable model | | N | Event (%) | Univariable model | | Multivariable model | |
|  |  |  | HR (95% CI) | p-value | HR (95% CI) | p-value |  |  | HR (95% CI) | p-value | HR (95% CI) | p-value |
| pT1-2N0 | 1108 | 88 (7.9) | 1 (ref) |  | 1 (ref) |  | 1108 | 44 (4.0) | 1 (ref) |  | 1 (ref) |  |
| pT3-4N0 | 246 | 42 (17.1) | 2.82 (1.94-4.08) | <.001 | 1.57 (1.05-2.35) | 0.028 | 246 | 32 (13.0) | 4.12 (2.61-6.52) | <.001 | 1.93 (1.15-3.23) | 0.013 |
| pT1-2N1 | 14 | 5 (35.7) | 5.43 (2.20-13.41) | <.001 | 7.09 (2.50-20.09) | <.001 | 14 | 4 (28.6) | 8.19 (2.93-22.88) | <.001 | 5.78 (1.65-20.24) | 0.006 |
| pT3-4N1 | 14 | 5 (35.7) | 5.49 (2.22-13.56) | <.001 | 5.04 (1.72-14.77) | 0.003 | 14 | 4 (28.6) | 8.36 (2.99-23.35) | <.001 | 5.26 (1.71-16.18) | 0.004 |

Adjusted for nuclear grade and necrosis in multivariable model of MFS

Adjusted for ASA, platelet, nephrectomy, nuclear grade, sarcomatoid differentiation, and necrosis in multivariable model of RFS

Adjusted for age, BMI, diabetes, ASA, hemoglobin, nuclear grade, and sarcomatoid differentiation in multivariable model of OS

Adjusted for age, BMI, hemoglobin, creatinine, nuclear grade, sarcomatoid differentiation, and necrosis in multivariable model of CSS

**Supplementary Table 3. Comparison of baseline characteristics between pathologic nodal positive and negative groups table (N=1832)**

|  | |  | Total | pTxN1 | pT1-4N0 | p-value |
| --- | --- | --- | --- | --- | --- | --- |
|  |  |  | (n=1832) | (n=52) | (n=1780) |  |
| Age at operation | mean±STD |  | 55.5±12.4 | 53.6±13.9 | 55.6±12.4 | 0.2549 |
| Gender | male | 1 | 1287 | 34 (65.4) | 1253 (70.4) | 0.4361 |
|  | female | 2 | 545 | 18 (34.6) | 527 (29.6) |  |
| Body mass index (kg/cm2) | mean±STD |  | 24.4±3.4 | 23.3±3.3 | 24.4±3.4 | 0.0213 |
| Diabetes | yes | 1 | 224 | 6 (11.5) | 218 (12.3) | 0.8755 |
| Hypertension | yes | 1 | 664 | 16 (30.8) | 648 (36.4) | 0.3998 |
| Chronic renal failure | yes | 1 | 36 | 1 (1.9) | 35 (2) | 0.2271 |
| ECOG | 0,1,2 | 1 | 1017 | 42 (80.8) | 975 (54.8) | 0.2543 |
|  | 3,4 | 2 | 48 | 0 (0) | 48 (2.7) |  |
| ASA | 1 | 1 | 738 | 11 (21.2) | 727 (40.8) | 0.232 |
|  | 2 | 2 | 787 | 22 (42.3) | 765 (43) |  |
|  | 3 | 3 | 97 | 2 (3.9) | 95 (5.3) |  |
|  | 4 | 4 | 2 | 0 (0) | 2 (0.1) |  |
| Hb | median (IQR) |  | 14 (12.6-15.1) | 12.8 (11.2-13.9) | 14 (12.6-15.1) | <.0001 |
| Platelet | median (IQR) |  | 232 (195-277) | 279.5 (216-321) | 231 (194-276) | 0.0007 |
| Creatinine | median (IQR) |  | 1 (0.9-1.12) | 0.95 (0.86-1.14) | 1 (0.9-1.12) | 0.4261 |
| Albumin | median (IQR) |  | 4.3 (4-4.5) | 4.1 (3.7-4.3) | 4.3 (4-4.5) | <.0001 |
| AST | median (IQR) |  | 21 (17-28) | 20 (14-25) | 21 (17-28) | 0.0504 |
| ALT | median (IQR) |  | 21 (15-31) | 16 (12-26) | 21 (15-31) | 0.0108 |
| MDRD GFR | median (IQR) |  | 72.4 (62.2-83.2) | 71.95 (59.55-86.1) | 72.4 (62.2-83.2) | 0.9798 |
| Tumor location | Lt | 1 | 968 | 24 (46.2) | 944 (53) | 0.505 |
|  | Rt | 2 | 842 | 28 (53.9) | 814 (45.7) |  |
|  | Bilat | 3 | 7 | 0 (0) | 7 (0.4) |  |
| Nephrectomy | Open surgery | 1 | 1256 | 38 (73.1) | 1218 (68.4) | 0.5314 |
|  | Laparoscopic | 2 | 561 | 14 (26.9) | 547 (30.7) |  |
| Operative Extent | partial | 1 | 593 | 4 (7.7) | 589 (33.1) | <.0001 |
|  | radical | 2 | 304 | 28 (53.9) | 276 (15.5) |  |
| pT | T1 | 1 | 1324 | 14 (26.9) | 1310 (73.6) | <.0001 |
|  | T2 | 2 | 191 | 11 (21.2) | 180 (10.1) |  |
|  | T3 | 3 | 301 | 26 (50) | 275 (15.5) |  |
|  | T4 | 4 | 16 | 1 (1.9) | 15 (0.8) |  |
| Histology | clear cell | 1 | 1500 | 32 (61.5) | 1468 (82.5) | 0.0019 |
|  | non-clear cell | 2 | 292 | 17 (32.7) | 275 (15.5) |  |
|  | mixed | 3 | 28 | 1 (1.9) | 27 (1.5) |  |
| Nuclear grade | grade 1-2 | 1 | 885 | 13 (25) | 872 (49) | 0.0041 |
|  | grade 3-4 | 2 | 761 | 28 (53.9) | 733 (41.2) |  |
| Sarcomatoid differentiation | yes | 1 | 32 | 5 (9.6) | 27 (1.5) | 0.0017 |
| Necrosis | yes | 1 | 76 | 16 (30.8) | 60 (3.4) | <.0001 |
| Lymphovascular invasion | yes | 1 | 65 | 18 (34.6) | 47 (2.6) | <.0001 |
| Capsular invasion | yes | 1 | 128 | 16 (30.8) | 112 (6.3) | <.0001 |

**Supplementary table 4. Comparison of survival prognoses between pathologic nodal positive and negative groups**

| **Subgroup dataset** |  | MFS | | | | | RFS | | | | |
| --- | --- | --- | --- | --- | --- | --- | --- | --- | --- | --- | --- |
|  | **N** | Event (%) | Univariable model | | Multivariable model | | Event (%) | Univariable model | | Multivariable model |  |
|  |  |  | HR (95% CI) | p-value | HR (95% CI) | p-value |  | HR (95% CI) | p-value | HR (95% CI) | p-value |
| **pN(0)** | 1780 | 94 (5.3) | 1 (ref) |  | 1 (ref) |  | 246 (13.8) | 1 (ref) |  | 1 (ref) |  |
| **pN(1)** | 52 | 4 (7.7) | 1.68 (0.62-4.57) | 0.3121 | 1.89 (0.66-5.42) | 0.2351 | 27 (51.9) | 5.14 (3.45-7.65) | <.0001 | 3.37 (2.06-5.53) | <.0001 |
| **Subgroup dataset** |  | OS | | | | | CSS | | | | |
|  | **N** | Event (%) | Univariable model | | Multivariable model | | Event (%) | Univariable model | | Multivariable model | |
|  |  |  | HR (95% CI) | p-value | HR (95% CI) | p-value |  | HR (95% CI) | p-value | HR (95% CI) | p-value |
| **pN(0)** | 1780 | 230 (12.9) | 1 (ref) |  | 1 (ref) |  | 145 (8.2) | 1 (ref) |  | 1 (ref) |  |
| **pN(1)** | 52 | 21 (40.4) | 3.60 (2.30-5.65) | <.0001 | 3.49 (2.05-5.93) | <.0001 | 16 (30.8) | 4.28 (2.54-7.19) | <.0001 | 3.26 (1.72-6.20) | 0.0003 |

| **Supplementary Table 5. Comparison of Baseline characteristics among patient staged pT2-4 with pN0, pN1, and pNx groups table (N=843)** | | | | | | | | |  |
| --- | --- | --- | --- | --- | --- | --- | --- | --- | --- |
|  | |  | Total | pNx | pN0 | pN1 | p-value |  |  |
|  |  |  | (n=843) | (n=335) | (n=470) | (n=38) |  |  |  |
| Age at operation | mean±STD |  | 57.6±12.3 | 59.4±12.2 | 56.6±12.2 | 54.3±13 | 0.0014 |  |  |
| Gender | male | 1 | 577 | 233 (69.6) | 318 (67.7) | 26 (68.4) | 0.8503 |  |  |
|  | female | 2 | 266 | 102 (30.5) | 152 (32.3) | 12 (31.6) |  |  |  |
| Body mass index (kg/cm2) | mean±STD |  | 24.2±3.6 | 24.3±3.7 | 24.2±3.6 | 23±3.6 | 0.1255 |  |  |
| Diabetes | yes | 1 | 115 | 53 (15.8) | 58 (12.3) | 4 (10.5) | 0.3025 |  |  |
| Hypertension | yes | 1 | 359 | 156 (46.6) | 190 (40.4) | 13 (34.2) | 0.0999 |  |  |
| Chronic renal failure | yes | 1 | 10 | 8 (2.4) | 1 (0.2) | 1 (2.6) | 0.1928 |  |  |
| ECOG | 0,1,2 | 1 | 718 | 332 (99.1) | 358 (76.2) | 28 (73.7) | 0.0182 |  |  |
|  | 3,4 | 2 | 15 | 2 (0.6) | 13 (2.8) | 0 (0.0) |  |  |  |
| ASA | 1 | 1 | 228 | 71 (21.2) | 150 (31.9) | 7 (18.4) | 0.4158 |  |  |
|  | 2 | 2 | 364 | 132 (39.4) | 214 (45.5) | 18 (47.4) |  |  |  |
|  | 3 | 3 | 42 | 12 (3.6) | 28 (6) | 2 (5.3) |  |  |  |
| Hb | median (IQR) |  | 13.4 (11.6-14.6) | 13.6 (11.9-14.55) | 13.25 (11.6-14.6) | 12.7 (10.6-14.2) | 0.1872 |  |  |
| Platelet | median (IQR) |  | 243 (202-303) | 231.5 (196.5-285.5) | 252 (204-311) | 274 (208-314) | 0.0026 |  |  |
| Creatinine | median (IQR) |  | 1 (0.84-1.2) | 0.98 (0.81-1.19) | 1 (0.9-1.2) | 1 (0.9-1.14) | 0.0518 |  |  |
| Albumin | median (IQR) |  | 4.2 (3.8-4.5) | 4.3 (3.9-4.6) | 4.2 (3.8-4.4) | 4 (3.6-4.3) | 0.0004 |  |  |
| AST | median (IQR) |  | 21 (17-27) | 21 (17-28) | 20 (16-27) | 21.5 (16-26.5) | 0.2126 |  |  |
| ALT | median (IQR) |  | 19 (13-29) | 20 (14-29) | 18 (12-29) | 19.5 (14-27.5) | 0.1904 |  |  |
| MDRD GFR | median (IQR) |  | 70.6 (59.8-83.3) | 73.4 (60.3-87.6) | 68.75 (59.4-78.8) | 70.4 (57.6-82.9) | 0.0105 |  |  |
| Tumor location | Lt | 1 | 410 | 157 (46.9) | 237 (50.4) | 16 (42.1) | 0.5534 |  |  |
|  | Rt | 2 | 417 | 169 (50.5) | 226 (48.1) | 22 (57.9) |  |  |  |
|  | Bilat | 3 | 3 | 2 (0.6) | 1 (0.2) | 0 (0) |  |  |  |
| Nephrectomy | Open surgery | 1 | 535 | 134 (40) | 370 (78.7) | 31 (81.6) | <.0001 |  |  |
|  | Laparoscopic | 2 | 295 | 194 (57.9) | 94 (20) | 7 (18.4) |  |  |  |
| Operative Extent | partial | 1 | 63 | 38 (11.3) | 25 (5.3) | 0 (0) | 0.117 |  |  |
|  | radical | 2 | 448 | 287 (85.7) | 140 (29.8) | 21 (55.3) |  |  |  |
| pT | T2 | 2 | 334 | 143 (42.7) | 180 (38.3) | 11 (29) | 0.3882 |  |  |
|  | T3 | 3 | 486 | 185 (55.2) | 275 (58.5) | 26 (68.4) |  |  |  |
|  | T4 | 4 | 23 | 7 (2.1) | 15 (3.2) | 1 (2.6) |  |  |  |
| Histology | clear cell | 1 | 684 | 267 (79.7) | 395 (84) | 22 (57.9) | 0.0042 |  |  |
|  | non-clear cell | 2 | 143 | 65 (19.4) | 65 (13.8) | 13 (34.2) |  |  |  |
|  | mixed | 3 | 9 | 3 (0.9) | 5 (1.1) | 1 (2.6) |  |  |  |
| Nuclear grade | grade 1-2 | 1 | 244 | 120 (35.8) | 116 (24.7) | 8 (21.1) | 0.2438 |  |  |
|  | grade 3-4 | 2 | 497 | 212 (63.3) | 266 (56.6) | 19 (50) |  |  |  |
| Sarcomatoid differentiation | yes | 1 | 49 | 22 (6.6) | 24 (5.1) | 3 (7.9) | 0.5835 |  |  |
| Necrosis | yes | 1 | 177 | 111 (33.1) | 54 (11.5) | 12 (31.6) | <.0001 |  |  |
| Lymphovascular invasion | yes | 1 | 108 | 56 (16.7) | 37 (7.9) | 15 (39.5) | <.0001 |  |  |
| Capsular invasion | yes | 1 | 290 | 191 (57.0) | 87 (18.5) | 12 (31.6) | <.0001 |  |  |

Supplementary table 6. **Comparison of survival prognoses according to nodal status and LN dissection in pT2-4 staged patients**

|  |  | MFS | | | | | RFS | | | | |
| --- | --- | --- | --- | --- | --- | --- | --- | --- | --- | --- | --- |
| **Group** | **N** | Event (%) | Univariable model | | Multivariable model | | Event (%) | Univariable model | | Multivariable model |  |
|  |  |  | HR (95% CI) | p-value | HR (95% CI) | p-value |  | HR (95% CI) | p-value | HR (95% CI) | p-value |
| nonLND | 335 | 26 (7.8) | 1 (ref) |  | 1 (ref) |  | 246 (13.8) | 1 (ref) |  | 1 (ref) |  |
| LND | 508 | 65 (12.8) | 1.63 (1.03-2.57) | 0.0373 | 2.33 (1.42-3.82) | 0.0008 | 182 (35.8) | 2.24 (1.67-3.00) | <.0001 | 1.21 (0.80-1.81) | 0.3653 |
| pNx | 335 | 26 (7.8) | 1 (ref) |  | 1 (ref) |  | 59 (17.6) | 1 (ref) |  | 1 (ref) |  |
| pN0 | 470 | 62 (13.2) | 1.65 (1.04-2.61) | 0.0343 | 2.36 (1.43-3.91) | 0.0008 | 159 (33.8) | 2.06 (1.53-2.78) | <.0001 | 1.19 (0.78-1.80) | 0.4183 |
| pN1 | 38 | 3 (7.9) | 1.32 (0.40-4.38) | 0.6455 | 2.01 (0.58-7.01) | 0.2744 | 23 (60.5) | 5.43 (3.35-8.80) | <.0001 | 2.69 (1.48-4.89) | 0.0012 |
|  |  | OS | | | | | CSS | | | | |
| **Group** | **N** | Event (%) | Univariable model | | Multivariable model | | Event (%) | Univariable model | | Multivariable model | |
|  |  |  | HR (95% CI) | p-value | HR (95% CI) | p-value |  | HR (95% CI) | p-value | HR (95% CI) | p-value |
| nonLND | 335 | 230 (12.9) | 1 (ref) |  | 1 (ref) |  | 145 (8.2) | 1 (ref) |  | 1 (ref) |  |
| LND | 508 | 139 (27.4) | 2.57 (1.77-3.73) | <.0001 | 1.81 (1.11-2.94) | 0.0172 | 106 (20.9) | 2.54 (1.68-3.86) | <.0001 | 1.79 (1.03-3.10) | 0.0379 |
| pNx | 335 | 35 (10.5) | 1 (ref) |  | 1 (ref) |  | 28 (8.4) | 1 (ref) |  | 1 (ref) |  |
| pN0 | 470 | 122 (26.0) | 2.38 (1.63-3.47) | <.0001 | 1.50 (0.92-2.46) | 0.106 | 92 (19.6) | 2.34 (1.53-3.57) | <.0001 | 1.51 (0.85-2.68) | 0.1592 |
| pN1 | 38 | 17 (44.7) | 5.70 (3.19-10.19) | <.0001 | 4.44 (2.24-8.82) | <.0001 | 14 (36.8) | 5.89 (3.1-11.19) | <.0001 | 4.38 (2.08-9.26) | 0.0001 |

**Supplementary Figure 1. Kaplan-Meier curves for (A) MFS, (B) RFS, (C) OS, and (D) CSS in relation to tumor histology among LN(+) patients**

| **(a)** | **(b)** |
| --- | --- |
| 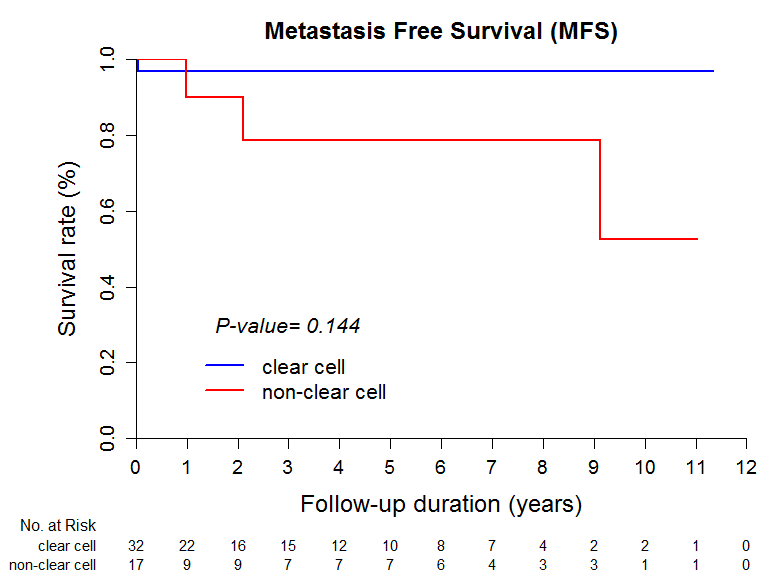 | 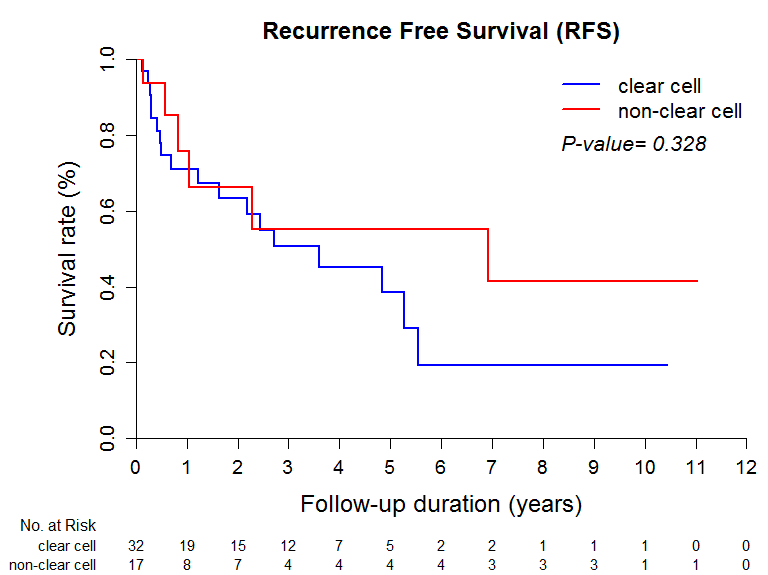 |

| **(c)** | **(d)** |
| --- | --- |
| 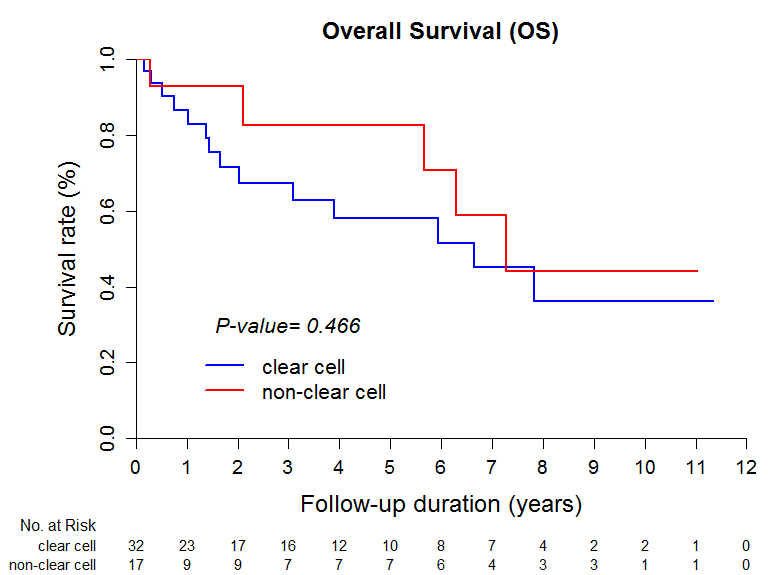 | 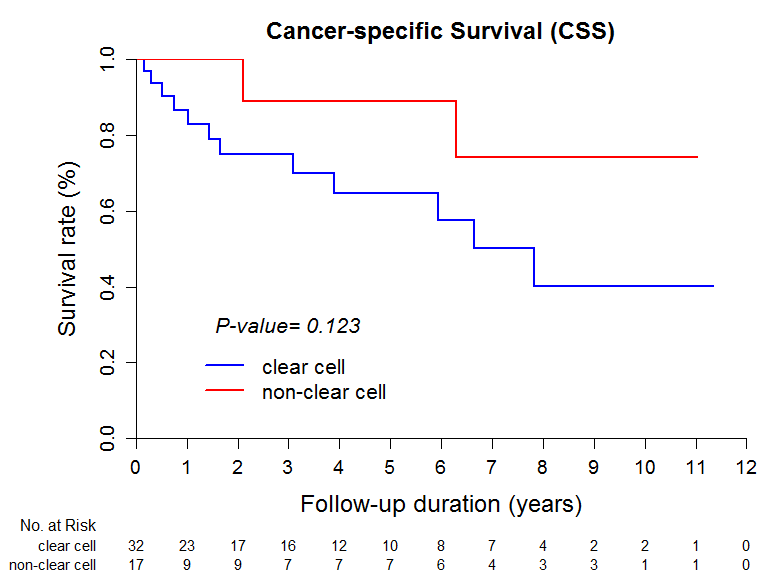 |

**Supplementary Figure 2. Kaplan-Meier curves for (A) MFS, (B) RFS, (C) OS, and (D) CSS in relation to T stage among LN(+) patients**

| **(a)** | **(b)** |
| --- | --- |
| 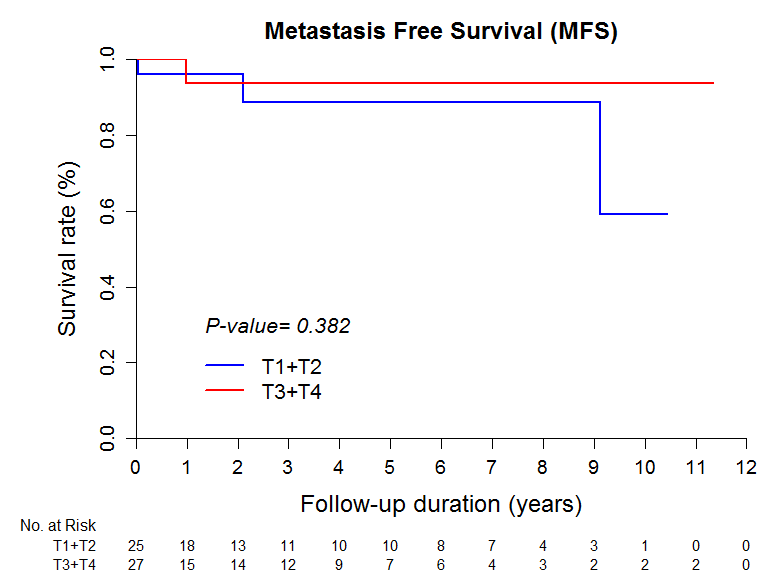 | 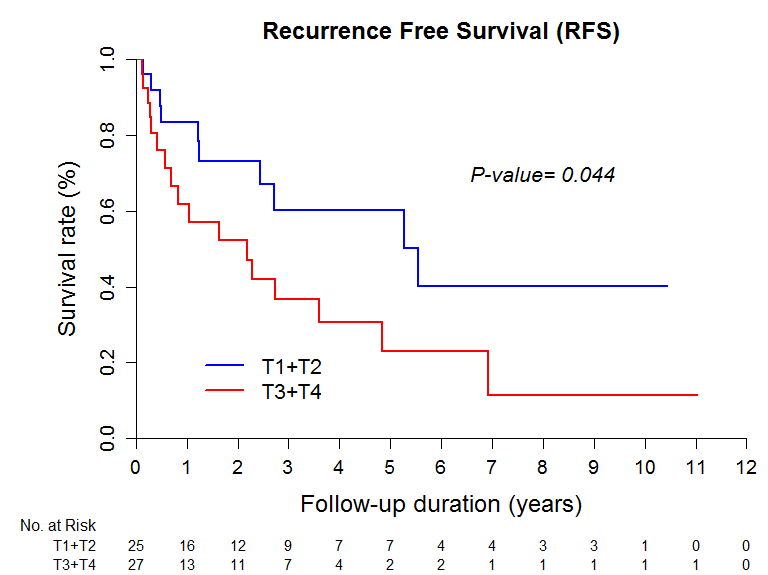 |

| **(c)** | **(d)** |
| --- | --- |
| 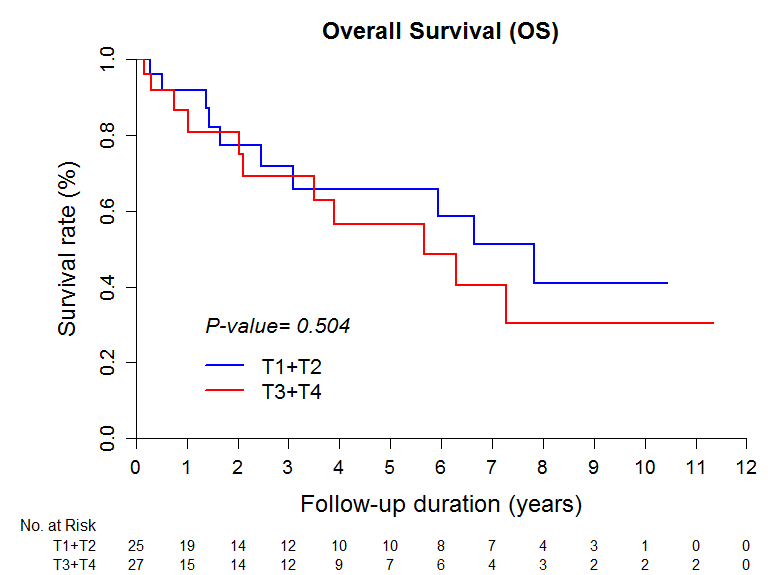 | 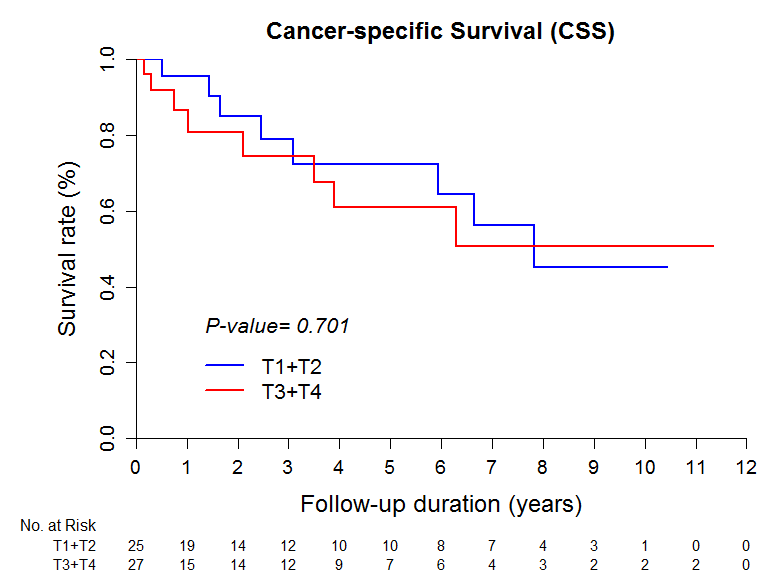 |
